# Predicting Bioactive Compounds in *Arbutus unedo* L. Leaves Using Machine Learning: Influence of Extraction Technique, Solvent Type, and Geographical Location

**DOI:** 10.3390/foods15060993

**Published:** 2026-03-11

**Authors:** Jasmina Lapić, Anica Bebek Markovinović, Nikolina Račić, Lana Vujanić, Marko Kostić, Dušan Rakić, Senka Djaković, Danijela Bursać Kovačević

**Affiliations:** 1 Faculty of Food Technology and Biotechnology, University of Zagreb, Pierottijeva 6, 10000 Zagreb, Croatia; 2 Faculty of Agriculture, University of Novi Sad, Trg Dositeja Obradovića 8, 21000 Novi Sad, Serbia; 3 Faculty of Technology, University of Novi Sad, Blvd. Cara Lazara 1, 21000 Novi Sad, Serbia

**Keywords:** strawberry tree leaves, extraction, geographical location, extraction solvent, polyphenols, machine learning

## Abstract

This study investigates the effects of extraction technique, solvent type, and geographical origin on the recovery of bioactive compounds from *Arbutus unedo* L. leaves collected from two Croatian islands (Vis and Mali Lošinj) and extracted using conventional, Soxhlet, and ultrasound-assisted extraction (UAE) with green solvents (distilled water, 70% ethanol, and ethyl acetate). Extracts were purified and characterized by thin-layer chromatography, column chromatography, and FTIR spectroscopy. Total phenols, hydroxycinnamic acids, flavonols, condensed tannins, and antioxidant capacity were quantified spectrophotometrically. Solvent type had the greatest influence, with 70% ethanol yielding the highest levels of bioactives and antioxidant capacity. Geographical origin significantly affected total phenolics and condensed tannins, with leaves from Vis outperforming those from Mali Lošinj. UAE was slightly more efficient than conventional and Soxhlet methods, particularly for thermolabile phenolics. Machine learning algorithms were applied as exploratory tools, using total phenols as a proxy variable to estimate selected bioactive compounds and antioxidant capacity based on extraction parameters. Decision Tree and Gradient Boosting models showed high goodness of fit within the experimental dataset (R^2^ > 0.91). These results support the potential of green extraction strategies combined with data-driven screening for the valorization of *A. unedo* leaf extracts, while highlighting the need for further validation prior to industrial application.

## 1. Introduction

*Arbutus unedo* L. (strawberry tree) is an evergreen species native to the Mediterranean region, traditionally valued for its fruits and leaves and increasingly investigated as a source of phenolic compounds. Previous studies have shown that *A. unedo* leaves contain diverse phenolic subclasses, including hydroxycinnamic acids, flavonoids, and condensed tannins, which contribute to their antioxidant capacity as measured by in vitro assays [1,2,3]. Rather than implying direct health effects, these properties are commonly assessed as indicators of bioactive potential relevant for food, nutraceutical, and cosmetic applications.

The phenolic composition of *A. unedo* leaves is known to vary considerably depending on environmental and agronomic factors. Geographical location, climate, and local growing conditions can influence plant secondary metabolism, leading to differences in phenolic accumulation and antioxidant capacity [4,5,6]. In parallel, extraction-related variables, particularly solvent polarity and extraction technique, play a major role in determining the efficiency and selectivity of phenolic recovery from plant matrices [7,8]. For phenolic-rich materials, hydroalcoholic solvents are frequently reported as the most effective, while alternative techniques such as ultrasound-assisted extraction (UAE) are often explored to improve mass transfer and reduce extraction time [9]. Conventional extraction techniques are simple and require minimal equipment, but they are time-consuming, consume large amounts of solvents and usually yield lower amounts of bioactive compounds. These methods are also less effective at extracting complex or thermally sensitive compounds [10]. Soxhlet extraction provides higher yields than conventional methods due to the prolonged exposure to the solvent and is therefore suitable for the extraction of both polar and non-polar compounds. However, it is time-consuming, energy-intensive, and carries the risk of thermal degradation for heat-sensitive compounds [11]. The UAE method, which uses ultrasonic waves to create cavitation and break the plant cell walls, improves the release of bioactive compounds. This method is faster, requires less solvent and better preserves thermally sensitive compounds, making it very efficient [12]. However, reported results are highly dependent on plant material, solvent system, and experimental design, making comparative, system-specific studies necessary.

In the case of *A. unedo* leaves, existing literature provides valuable data on phenolic composition and antioxidant activity, yet comparisons across studies are often complicated by differences in solvents, extraction conditions, and analytical endpoints. Several studies have investigated individual extraction approaches or solvent systems, but direct comparisons across multiple extraction techniques, solvent types, and geographical origins within a single, factorially designed experiment remain limited. Accordingly, the present work does not aim to claim absolute novelty, but rather to contribute comparative data generated under controlled and internally consistent conditions.

In recent years, data-driven approaches have been increasingly introduced into food and natural product research to support experimental interpretation and process screening. Machine learning (ML) methods, in particular, can be useful for exploring relationships among measured variables and identifying patterns within multivariate datasets [13]. While ML is often associated with large datasets, simpler “shallow learning” models can also be informative when applied cautiously to small, structured, factorial datasets. In such contexts, ML should be viewed as an exploratory and supportive tool rather than a means of producing broadly generalizable predictive models. When combined with appropriate validation strategies, ML can assist in evaluating whether easily accessible parameters, such as total phenolic content can serve as proxies for more analytically demanding measurements within a defined experimental space [13,14,15].

Therefore, the primary aim of this study was to evaluate the influence of geographical origin, extraction technique, and solvent type on phenolic indices (total phenolics, hydroxycinnamic acids, flavonols, and condensed tannins) and antioxidant capacity (FRAP and ABTS assays) of *A. unedo* leaf extracts. A secondary aim was to explore the use of machine learning models as a complementary analytical tool for assessing internal relationships among these measured parameters within a small, factorial dataset. FTIR spectroscopy was applied as a qualitative technique to support the interpretation of extract composition by identifying dominant functional groups and chemical classes, rather than for compound-level confirmation. Collectively, this approach is intended to support comparative evaluation and preliminary screening of extraction strategies for *A. unedo* leaves, while explicitly acknowledging methodological and interpretative limitations.

## 2. Materials and Methods

### 2.1. Plant Material

Leaves of *A. unedo* were collected in October 2024 from the Croatian islands Vis and Mali Lošinj. Upon delivery to the laboratory, the fresh leaves were air-dried under ambient room-temperature conditions for eight days. Once fully dried, the leaves were crushed using a hand mixer (Robert Bosch Hausgeräte GmbH, Munich, Germany) and stored at room temperature in hermetically sealed dark glass containers until extraction procedures were performed.

### 2.2. Extraction of Bioactive Antioxidants from A. unedo Leaves

The experiment followed a full factorial design with 2 cultivation locations (LC) × 3 extraction techniques (TE) × 3 extraction solvents (ES), resulting in 18 unique extraction conditions. For each LC × TE × ES condition, two independent extraction replicates were prepared as separate extracts, yielding 36 independent extracts in total.

The solvent composition, solid–liquid ratio, and extraction times were selected based on established extraction principles and prior optimization studies for phenolic-rich plant matrices. A plant material-to-solvent ratio of 1:10 was applied for conventional and Soxhlet extraction to ensure adequate mass transfer while maintaining methodological comparability across solvents, as commonly reported in solid–liquid extraction protocols [9,16]. The use of 70% (*v*/*v*) ethanol was chosen due to its proven efficiency in extracting a broad range of phenolic compounds by combining the polarity of water with the solubilization capacity of ethanol [9,16]. An extraction time of 1 h was selected as a compromise between extraction efficiency and minimization of thermal degradation during prolonged heating [10]. For ultrasound-assisted extraction, a reduced extraction time (10 min) and higher solvent-to-solid ratio were employed to exploit cavitation-induced cell disruption and enhance phenolic release while limiting thermal and oxidative stress, in accordance with previously optimized UAE conditions for bioactive compounds [12].

#### 2.2.1. Conventional Extraction (CE)

Conventional extraction was carried out using different solvents; distilled water, 70% ethanol and ethyl acetate at the boiling point of the solvent itself with a ratio of plant material to solvent of 1:10. Extraction was carried out in an apparatus for refluxing and heating was carried out using a heating mantle. Crushed leaves (5 g) and extraction solvent (50 mL) were added to a round bottom flask and refluxing was being carried out for 1 h. After the extraction is complete, the extract is cooled and filtered using filter paper. Ten mL of the total volume of each filtrate was separated, necessary for determining the phenolic profile. The remaining filtrate was evaporated to dryness on a rotary evaporator, resulting in samples of different colors and masses and a characteristic odor (Table 1).

#### 2.2.2. Soxhlet Extraction (SE)

The extraction solvent, distilled water, and 70% ethanol or ethyl acetate (50 mL) were added to a round bottom flask while the plant sample of crushed leaves (5 g) was weighed into a Soxhlet thimble extractor and placed in the extraction apparatus. A heating mantle was used to reflux the mixture for an extraction time of 1 h. After the extraction time, the extract solution was allowed to cool to room temperature and then filtered through filter paper. Ten mL of the filtrate was separated for determination phenolic profile and the residue was concentrated to dryness using a rotary evaporator, yielding samples of masses (Table 2).

#### 2.2.3. Ultrasound-Assisted Extraction (UAE)

An ultrasonic processor UP400St (Hielscher Ultrasonics GmbH, Teltow, Germany) was utilized for UAE. The extraction of bioactive compounds (BACs) followed a modified protocol from the literature [17]. For the procedure, 1 g of dried, ground *A. unedo* leaves was placed into a glass beaker, and 80 mL of the selected extraction solvent was added. The extraction mixtures were processed under the following conditions: a 22 mm probe diameter, 100% amplitude, pulse mode (0.5 s on/0.5 s off; 50% duty cycle), and a 10 min extraction time, resulting in a total energy input of 120 kJ (Table 3). During UAE, temperature was monitored to control ultrasound-induced heating. All extractions were performed in a cold-water bath supplemented with ice. Under these conditions, the mean extraction temperature was 45.2 °C, with no abrupt temperature increase observed during the 10 min sonication at 100% amplitude. The obtained extracts were then filtered into 100 mL volumetric flasks and diluted to the mark with the same extraction solvent.

Gravimetric yield was not determined for UAE because extracts were diluted to a fixed volume for analytical standardization and not evaporated to dryness. As extraction efficiency was assessed based on bioactive compound recovery per gram of dry plant material, total yield was not considered essential for method comparison.

### 2.3. Purification by Using Preparative Thin Column-Chromatography (CC)

TLC was performed on leaf extracts obtained by CE and SE to determine the composition of the fractions. Silica gel 60 F254 (Merck, Darmstadt, Germany) was used as the stationary phase, and a solvent mixture of dichloromethane: ethanol (10:1, *v*/*v*) was used as the mobile phase. The plates were developed in a pre-saturated chamber and visualized under UV light at 254 nm.

In all extracts from the island of Vis and the island of Mali Lošinj, regardless of the extraction technique (CE or SE) or the solvent used (water, 70% ethanol, ethyl acetate), four dominant fractions were observed on TLC. The *R*_f_ values for all dominant fractions in all extracts for each solvent and island are listed in the Supplementary Materials as Tables S3 and S4. Accordingly, all extracts obtained by CE and SE were pooled separately for each locality (Vis and Mali Lošinj) and subjected to column chromatography for fractionation and FTIR analysis.

The samples were separated on a glass column packed with silica gel, 0.063–0.2 mm. Owing to the low solubility of the sample in the mobile phase, the concentrated extract was homogenized with a small amount of silica gel and placed on top of the column. Elution was performed with solvents of increasing polarity, resulting in the isolation of four dominant fractions from each sample (Table 4).

The relative mass share (%) of each pooled fraction is summarized in Table 4, calculated as the percentage of the total mass of isolated dominant components (∑ fractions = 100%).

### 2.4. Characterization by IR Spectroscopy

Fourier-transform infrared spectroscopy (FTIR) was employed as a qualitative characterization technique to identify major functional groups and dominant chemical classes present in the purified fractions of *A. unedo* leaf extracts. The purpose of this analysis was to support the phytochemical interpretation of the extracts and to complement the spectrophotometric results, rather than to provide quantitative input for the statistical or machine learning analyses.

The IR spectra were recorded for KBr pellets with a Bruker ALPHA-FT-IR spectrophotometer and appropriate computer software. The obtained IR spectra were scanned from wave number ranging from 4000 to 500 cm^−1^ with a resolution of 4 cm^−1^.

L1_ML: IR (KBr), ν_max_/cm^−1^ = 3049 (w., C–H, aromatic) 2920, 2851 (s., C–H), 1733 (m., C=O), 1378 (w., CH_2_), 1174, 1093 (m., C–O, C–C).

L2_ML: IR (KBr), ν_max_/cm^−1^ = 3416 (b. m., O–H), 3036 (w., C–H, aromatic), 2920, 2855 (s., C–H), 1735 (m., C=O), 1378 (w., CH_2_), 1168, 1093 (w., C–O, C–C).

L3_ML: IR (KBr), ν_max_/cm^−1^ = 3397 (b. m., O–H), 3045 (w., C–H, aromatic), 2922, 2851 (s., C–H), 1720, 1689 (w., C=O), 1460 (w.), 1381 (w., CH_2_), 1189, 1040 (w., C–O, C–C).

L4_ML: IR (KBr), ν_max_/cm^−1^ = 3379 (b. m., O–H), 3045 (w., C–H, aromatic), 2920, 2851 (s., C–H), 1721, 1688 (m., C=O), 1461 (m.) and 1380 (w., CH_2_), 1187, 1041 (w., C–O, C–C).

L1_V: IR (KBr), ν_max_/cm^−1^ = 3049 (w., C–H, aromatic) 2949, 2851 (s., C–H), 1733 (m., C=O), 1460, 1378 (m., CH_2_), 1170, 987 (m., C–O, C–C).

L2_V: IR (KBr), ν_max_/cm^−1^ = 3395 (b. m., O–H), 3036 (w., C–H, aromatic), 2924, 2851 (s., C–H), 1737, 1713 (m., C=O), 1462, 1378 (m., CH_2_), 1162, 983 (w., C–O, C–C).

L3_V: IR (KBr), ν_max_/cm^−1^ = 3393 (b. m., O–H), 3045 (w., C–H, aromatic), 2922, 2851 (s., C–H), 1709, 1689 (w., C=O), 1460, 1378 (w., CH_2_), 1034, 983 (w., C–O, C–C).

L4_V: IR (KBr), ν_max_/cm^−1^ = 3365 (b. m., O–H), 3045 (w., C–H, aromatic), 2924, 2853 (s., C–H), 1690 (m., C=O), 1454 1382 (w., CH_2_), 1044, 995 (w., C–O, C–C).

### 2.5. Determination of Bioactive Compounds and Antioxidant Capacity in Extracts from A. unedo Leaves

All measurements were performed using an LLG-uniSPEC 2 spectrophotometer (Lab Logistics Group GmbH, Meckenheim, Germany). For each extract, analytical measurements were performed in triplicate, and the three readings were averaged to obtain a single extract-level value for each response variable. These extract-level means were used as the input for statistical analyses (e.g., ANOVA and correlation). Triplicate readings were not treated as independent observations in order to avoid pseudoreplication; replicate-level variability was used only to report measurement dispersion (e.g., SD and CV) where appropriate.

#### 2.5.1. Determination of Total Phenolic Content (TPC)

A modified Folin–Ciocalteu method, as described in the literature, was used to determine the total phenolic content (TPC) [18]. Briefly, 400 µL of diluted extract (the water extract was diluted 25-fold, the 70% ethanol extract was diluted 20-fold, and the ethyl acetate extract was diluted 10-fold), 400 µL of Folin–Ciocalteu reagent (5-fold dilutetd), and 4 mL of 7.5% sodium carbonate solution were combined. The reaction mixture was left to stand at room temperature for 20 min. Subsequently, the absorbance was measured at 725 nm. TPC was calculated based on a calibration curve constructed with gallic acid solutions (10–250 mg L^−1^). The results were expressed as milligrams of gallic acid equivalent (GAE) per gram of sample.

#### 2.5.2. Determination of Total Hydroxycinnamic Acids (HCA) and Total Flavonols (FL)

A modified spectrophotometric method was employed to quantify hydroxycinnamic acids (HCA) and flavonols (FL) [19]. A 250 µL aliquot of appropriately diluted extract was mixed with 250 µL of Solution 1 (1 g L^−1^ HCl in 96% ethanol) and 4.55 mL of Solution 2 (2 g L^−1^ HCl in distilled water). The mixture was shaken for 10 s and then incubated at room temperature for 30 min in dark. Afterwards, absorbance at 320 nm was measured for HCA and 360 nm for FL. A blank sample was prepared using the same procedure, replacing the extract with the extraction solvent. HCA concentrations were calculated using a calibration curve generated with chlorogenic acid standard solution (10–600 mg L^−1^), while FL concentrations were determined using a calibration curve prepared with quercetin at the same concentration range. Results were expressed as milligrams of chlorogenic acid equivalents (CAE) per g of sample for HCA and milligrams of quercetin equivalents (QE) per g of sample for FL.

#### 2.5.3. Determination of Condensed Tannins (CT)

A modified spectrophotometric method from the literature was applied to determine the condensed tannin (CT) content [20]. Briefly, 2.5 mL of 1% vanillin solution, 2.5 mL of 25% H_2_SO_4_ solution, and 1 mL of appropriately diluted extract were combined in a glass test tube and mixed thoroughly. The mixture was left to stand at room temperature for 10 min. Absorbance was measured at 500 nm. A blank sample was prepared following the same procedure, substituting the extract with the extraction solvent. A calibration curve was created using catechin solutions (10–120 mg L^−1^). Results were expressed as milligrams of catechin equivalents (CA) per gram of dried leaf sample.

#### 2.5.4. Determination of In Vitro Antioxidant Capacity (AOC)

##### Ferric Reducing Antioxidant Power Assay (FRAP)

The antioxidant capacity was evaluated using the FRAP method [21]. To prepare the FRAP reagent, 50 mL of 0.3 M acetate buffer (pH 3.6) was mixed with 5 mL of 10 mM tripyridyltriazine (TPTZ) solution dissolved in 40 mM HCl, and 5 mL of 20 mM ferric chloride (FeCl_3_) solution. Briefly, 4.5 mL of the FRAP reagent was combined with 600 µL of the appropriately diluted extract in glass tubes. The mixture was homogenized for 1 min using a vortex shaker (Grant Instruments Ltd., Cambridge, UK) and incubated in a water bath at 37 °C/10 min. The absorbance was measured at 593 nm. A blank sample was prepared using the same procedure, except the extract was replaced with the extraction solvent. A calibration curve was created with varying concentrations of Trolox solution (10–150 µM), and the results were expressed as µmol Trolox equivalents per g of sample.

##### 2,2-Azino-bis-3-ethylbenzothiazoline-6-sulphonic Acid Assay (ABTS)

The antioxidant capacity was also evaluated using the ABTS method [22]. To prepare the ABTS reagent, 88 µL of potassium persulfate (K_2_S_2_O_8_) was mixed with 5 mL of ABTS solution on the first day, resulting in a final potassium persulfate concentration of 2.45 mmol L^−1^. This solution served as the ABTS^+^ reagent and stored in the dark for 12–16 h. On the second day, a 1% ABTS solution was prepared by diluting the ABTS^+^ reagent, considering it as a 100% solution, with 96% ethanol. The absorbance of the diluted ABTS solution was measured at 734 nm using 96% ethanol as the blank, with an expected absorbance of 0.734 ± 0.02. Firstly, 240 µL of the appropriately diluted extract was pipetted into a glass test tube, followed by the addition of 3 mL of ABTS solution. The absorbance was measured 10 min after incubation at 37 °C at 734 nm using. A blank test was performed using 96% ethanol. A calibration curve was created using Trolox solutions (25–300 µM), and the results were expressed as µmol Trolox equivalents per g of sample.

### 2.6. Statistical Analysis

Descriptive statistics that included mean values, minimum and maximum values, standard deviations, coefficients of variation, and confidence intervals were used to accurately describe and analyze data sets. For each extract, analytical measurements were performed in triplicate; these analytical replicates were not treated as independent observations but were averaged to obtain a single value per response variable for that extract. Replicate-level results were used only to describe measurement dispersion (e.g., SD/CV).

An analysis of variance (ANOVA) was used to determine if there were statistically significant differences in the dependent variables between groups defined by the independent variables. The ANOVA was conducted by Tukey’s Honest Significant Difference (HSD) test to identify specific group differences post hoc. The level of statistical significance for all tests was set at *p* ≤ 0.05, ensuring robust inference. The analysis was carried out using Statistica software (version 14.1). ANOVA assumptions were evaluated using residual diagnostics. Normality of residuals was assessed using Shapiro–Wilk tests and Q–Q plots. Deviations from normality were observed for TPC (*p* = 0.0006), HCA (*p* = 0.0037) and FRAP (*p* = 0.0148), whereas FL (*p* = 0.884), CT (*p* = 0.0537) and ABTS (*p* = 0.101) did not show strong evidence against normality. Homoscedasticity was assessed primarily using residuals-versus-fitted and scale–location plots; formal variance tests across all factorial cells are unstable with very small cell sizes, so residual plots were prioritized.

For each response variable (TPC, HCA, FL, CT, FRAP, ABTS), a factorial ANOVA was fitted using cultivation location (LC), extraction technique (TE), and extraction solvent (ES) as fixed factors, including all two-way interaction terms: Y = μ+ LC + TE + ES + LC × TE + LC × ES + TE × ES + ε, where Y is the response and ε is the residual error term. Statistical significance was assessed at α = 0.05. When significant main effects were detected, Tukey’s HSD post hoc test was applied for pairwise comparisons.

Where two-way interactions were significant, main effects were interpreted cautiously and post hoc comparisons were performed within the relevant factor combinations. The three-way interaction (LC × TE × ES) was not included/was not interpreted in the primary analysis to avoid over-parameterization given the limited replication per cell (two extracts per LC × TE × ES condition).

In addition, ANOVA was employed to assess the contribution of individual main effects to the total variability of the dependent variables (interpreted as marginal contributions in the presence of interactions). This was achieved by proportionally partitioning the sum of squares (SS) to quantify the relative influence of each factor. When degrees of freedom for the observed effects differed, the contribution was calculated using the mean square errors (MSE), allowing for precise comparison across effects. Pearson’s coefficient of correlation (r) was calculated to determine the linear correlation between the observed parameters. Standard deviation (SD) and coefficient of variation (CV) were used to describe the variability of the measured values across samples/experimental conditions. In contrast, confidence intervals (CI) quantify the uncertainty of the estimated mean and therefore reflect the precision of the mean rather than the spread of individual observations. Thus, high SD/CV indicates high data variability, whereas a wide CI indicates lower confidence in the mean estimate (typically due to small n or high variability).

Variability in this study can be partitioned into (i) biological variability associated with cultivation location (LC), (ii) process variability driven by extraction parameters (TE and ES), and (iii) analytical variability from triplicate measurements within each extract. ANOVA quantifies the contribution of biological and process factors to between-extract variability, while analytical triplicates were averaged and used only to report measurement dispersion.

### 2.7. Machine Learning-Based Linear Modeling

In this study, machine learning (ML) techniques were employed to develop predictive models for key bioactive compounds present in *A. unedo* leaf extracts. The primary objective was to model each target compound (HCA, FL, CT, ABTS, and FRAP) individually, using TPC as the principal numerical predictor, in combination with categorical variables describing extraction conditions (cultivation location, extraction technique, and extraction solvent).

The rationale behind this approach was to evaluate whether TPC, a parameter that is relatively simple and inexpensive to quantify, could serve as a reliable proxy for estimating other, analytically more demanding antioxidant and bioactive properties. If accurate and robust ML models can be constructed using TPC and basic categorical descriptors, this would enable researchers to predict HCA, FL, CT, ABTS, and FRAP values without performing time-consuming and costly chemical assays for each. This approach may serve as a preliminary screening approach to support rapid comparative assessment of extraction conditions, rather than as a substitute for detailed chemical analyses.

#### 2.7.1. Shallow Learning Algorithms in Feature Prediction

The dataset containing TPC along with categorical factors was imported into Python and processed using the Pandas library. The ML dataset is small (n = 36 extracts), which increases the risk of overfitting, particularly when comparing multiple algorithms. Therefore, model performance estimates should be interpreted as exploratory within this experimental space rather than as evidence of broad generalizability.

The ML unit of analysis was the independent extract replicate. Thus, each row corresponds to one extract (18 LC × TE × ES conditions × 2 independent extraction replicates = 36 observations). Triplicate analytical readings were averaged prior to modeling to obtain one response value per extract. No extraction conditions were excluded and there were no missing LC × TE × ES combinations in the final dataset. Abbreviations used in this section: LC—cultivation location; TE—extraction technique; ES—extraction solvent; TPC—total phenolic content; HCA—hydroxycinnamic acids; FL—flavonols; CT—condensed tannins; FRAP—ferric reducing antioxidant power; ABTS—ABTS radical scavenging assay.

The predictor set included one numerical feature (TPC) and three categorical descriptors (LC, TE, and ES). Categorical variables were one-hot-encoded to create binary indicator features, while TPC was standardized using z-scores. To avoid data leakage, all preprocessing steps were implemented within a scikit-learn Pipeline/ColumnTransformer and were fitted only on the training data. During cross-validation, preprocessing was re-fitted within each training fold and then applied to the corresponding validation fold.

To assess the added value of more flexible algorithms under the strong correlation structure observed among TPC and several target variables, baseline linear models were also included, namely ordinary least squares Linear Regression and regularized linear models (Ridge, Lasso, and ElasticNet) using the same predictors (TPC + one-hot-encoded LC/TE/ES). Baseline performance is reported in the Supplementary Material (Table S1).

The dataset was split into a training set (80%) and a held-out test set (20%) using a fixed random seed (random_state = 42). Model selection (and any tuning, where applicable) was performed only on the training set using 5-fold cross-validation. The selected model was then refit on the full training set and evaluated once on the held-out test set, which was not used during model selection. This validation strategy was adopted to ensure a leakage-free and reproducible evaluation workflow.

**Figure 1:**
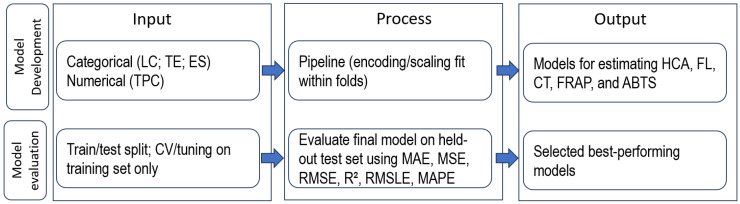
Workflow of the shallow learning analysis. Categorical descriptors (LC, TE, ES) and the numerical predictor TPC were used as model inputs. Preprocessing (encoding/scaling) was performed within a leakage-free pipeline, fitted only on the training folds during cross-validation. Model selection/tuning was conducted on the training set only, whereas final performance was evaluated on the held-out test set using MAE, MSE, RMSE, R^2^, RMSLE, and MAPE.

Model performance was assessed using MAE, MSE, RMSE, R^2^, RMSLE, and MAPE. Error metrics were reported in the original units of each response variable, whereas R^2^ was unitless. The workflow of the shallow learning analysis is presented in Figure 1.

External validation on an independent dataset was not available. In addition, structured validation to test transferability across cultivation locations (e.g., leave-one-location-out) was not performed. Consequently, the reported results reflect internal performance only, and location-to-location generalization cannot be claimed. All analyses were performed in Python (version 3.10), and the software versions used are reported in the Supplementary Material.

#### 2.7.2. Model Accuracy Evaluation

Several metrics were used to quantify the model’s predictive ability, and their explanations can be found in the literature [23]:

Mean Absolute Error (MAE)

(1) $$MAE=\frac{1}{n}\sum_{i=1}^{n}\left|y_{i}-{\hat{y}}_{i}\right|$$

Mean Squared Error (MSE)

(2) $$MSE=\frac{1}{n}\sum_{i=1}^{n}{\left(y_{i}-{\hat{y}}_{i}\right)}^{2}$$

Root Mean Squared Error (RMSE)

(3) $$RMSE=\sqrt{\frac{1}{n}\sum_{i=1}^{n}{\left(y_{i}-{\hat{y}}_{i}\right)}^{2}}$$

Coefficient of Determination (R^2^)

(4) $$R^{2}=1-\frac{\sum_{i=1}^{n}{\left(y_{i}-{\hat{y}}_{i}\right)}^{2}}{\sum_{i=1}^{n}{\left(y_{i}-\bar{y}\right)}^{2}}$$

Root Mean Square Logarithmic Error (RMSLE)

(5) $$RMSLE=\sqrt{\frac{1}{n}\sum_{i=1}^{n}{\left(log\left(y_{i}+1\right)-log\left({\hat{y}}_{i}+1\right)\right)}^{2}}$$

Mean Absolute Percentage Error (MAPE)

(6) $$MAPE=\frac{100}{n}\sum_{i=1}^{n}\left|\frac{y_{i}-{\hat{y}}_{i}}{y_{i}}\right|$$

Time Taken (TT) = Total Time taken for model training and evaluation in seconds

## 3. Results and Discussion

### 3.1. Fourier-Transform Infrared Spectroscopy (FTIR) Characterization of A. unedo Leaf Extracts

Extraction is the first crucial step in teh isolation of bioactive compounds from plant material. This process requires a tailored approach for each plant species and involves selecting a suitable solvent, as well as determining the optimal temperature and duration of extraction, all of which can significantly impact the efficiency of the process. The extraction technique directly influences the concentration and proportion of bioactive compounds in the extract, as the extraction conditions affect the stability and potential degradation of these compounds [24]. In this study, two conventional extraction techniques—reflux heating and Soxhlet extraction—were used alongside ultrasound-assisted methods as non-conventional approaches for the isolation of bioactive compounds.

Conventional solid–liquid extraction (CE) by reflux heating was performed using dry, crushed *A. unedo* leaves. To evaluate the influence of solvent polarity on the bioactive compound content, three solvents were used: distilled water, 70% ethanol, and ethyl acetate. Following extraction, the solvents were evaporated using a rotary evaporator, yielding solid extracts of varying colors, ranging from green to dark brown. For leaves from the Mali Lošinj area, the highest mass of evaporated plant extract was obtained with 70% ethanol (2067.7 ± 2.50 mg), while the lowest mass was recorded with ethyl acetate (65.00 ± 2.00 mg). A similar pattern was observed for leaf extracts from the island of Vis, where 70% ethanol yielded the highest extract mass (2663.00 ± 2.00 mg), and ethyl acetate produced the lowest (87.00 ± 3.00 mg). Soxhlet extraction (SE) was also conducted on the leaves using the same three solvents: distilled water, 70% ethanol and ethyl acetate. For leaves from Mali Lošinj, the highest mass of evaporated extract was again obtained with 70% ethanol (1512.33 ± 5.49 mg), whereas ethyl acetate yielded the lowest (75.07 ± 1.13 mg). For leaf extracts from the island of Vis, 70% ethanol produced the highest extract mass (1345.70 ± 6.03 mg), while similar low masses were obtained with ethyl acetate (46.33 ± 2.52 mg).

A comparison of the masses of isolated bioactive constituents extracted from the leaves using CE and SE shows that the largest mass was obtained with 70% ethanol as the extraction solvent. This aligns with published data indicating that binary solvent systems extract more phenolic compounds than monosystems (e.g., water, ethyl acetate, or pure ethanol) [25]. Binary solvent systems are more effective at extracting phenolic compounds than mono-solvent systems because they combine the solvent properties of their individual components. Binary systems offer a broader polarity spectrum, enabling them to solubilize both hydrophilic and hydrophobic compounds. Water’s high polarity facilitates the extraction of hydrophilic phenolics, while ethanol (or other organic solvents) reduces overall polarity, allowing for the dissolution of moderately polar and non-polar phenolics [16]. In addition, the presence of water enhances the penetration of the solvent into plant matrices by swelling plant cell walls, while ethanol disrupts lipid membranes, aiding the release of phenolic compounds. This complementary action increases extraction efficiency [26,27].

A slightly higher mass of extracted constituents was achieved with water compared to ethyl acetate. This is likely because water, being a more polar solvent with a higher boiling point, is more effective at extracting polar constituents. The mass of isolated constituents obtained via SE was significantly lower than that obtained through CE by reflux heating. This observation may be related to methodological differences between Soxhlet and conventional extraction, which have been reported in the literature to influence extraction efficiency. However, since temperature profiles were not directly monitored in the present study, definitive conclusions regarding temperature-driven effects cannot be drawn [28].

The leaf extracts from both areas were purified using column chromatography, resulting in four dominant fractions. During this process, the polarity of the eluent was gradually increased, with pure ethanol used as the final eluent. For the extracts from the island of Mali Lošinj, the largest yield was obtained in the third fraction, using dichloromethane and ethanol as the eluent in a 10:1 ratio, with a mass of 219.5 mg. In contrast, for the leaf extracts from the island of Vis, the highest yield was obtained in the fourth fraction, where ethanol alone was used as the eluent, yielding 372.2 mg.

**Figure 2:**
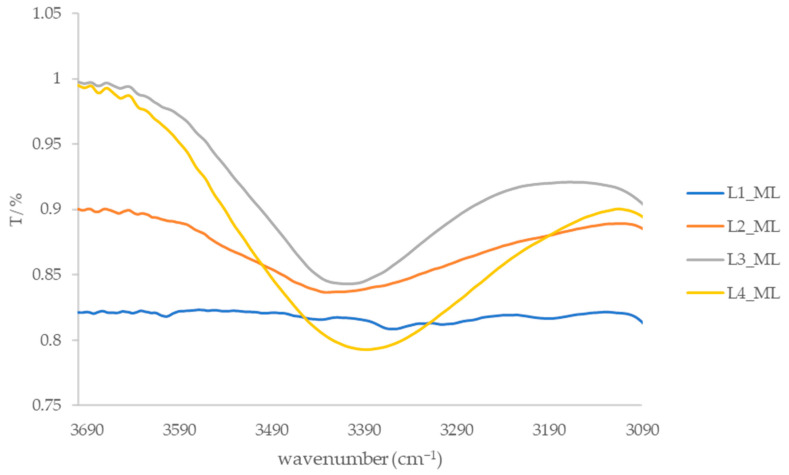
IR spectra of leaf fractions from the island of Mali Lošinj in the range of 3700–3090 cm^−1^.

**Figure 3:**
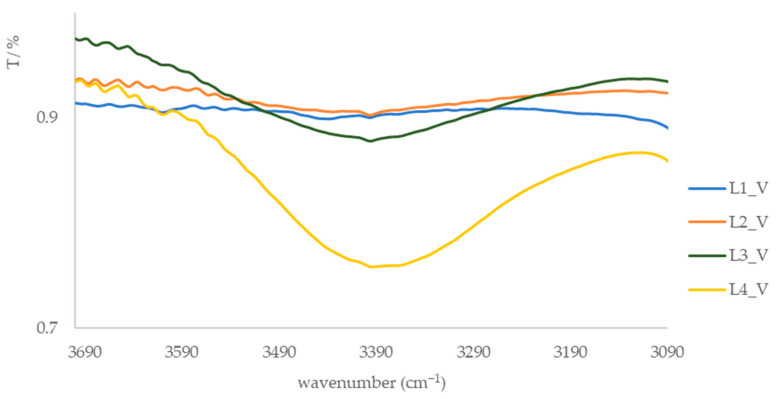
IR spectra of leaf fractions from the island of Vis in the range of 3700–3090 cm^−1^.

After extraction using the CE and SE methods and subsequent purification of the extracts via column chromatography, four dominant fractions (L1_ML to L4_ML and L1_V to L4_V) were isolated, and their IR spectra were recorded (Figures S1–S8). The samples were prepared and analyzed as KBr pellets. In the IR spectra of all fractions, an absorption band around 3040 cm^−1^ is observed, corresponding to the stretching vibration of aromatic and ethene =C–H bonds. Additional absorption bands at 2920 cm^−1^ and around 2851 cm^−1^ are attributed to the stretching frequencies of C–H bonds. A broad absorption band of medium to weak intensity, visible in the spectra of fractions L2, L3, and L4 (3416–3365 cm^−1^, Figure 2 and Figure 3), is associated with hydroxyl groups, likely belonging to phenolic compounds. This spectral feature is consistent with the presence of hydroxyl-containing compound classes, such as phenolic compounds and carboxylic acid derivatives, as indicated by carbonyl-related absorption bands in the 1710 cm^−1^ region.

**Figure 4:**
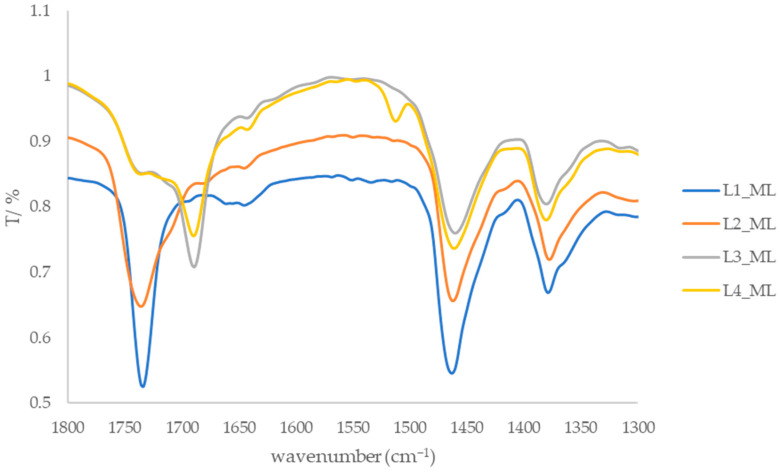
IR spectra of leaf fractions from the island of Mali Lošinj in the range of 1800–1300 cm^−1^.

**Figure 5:**
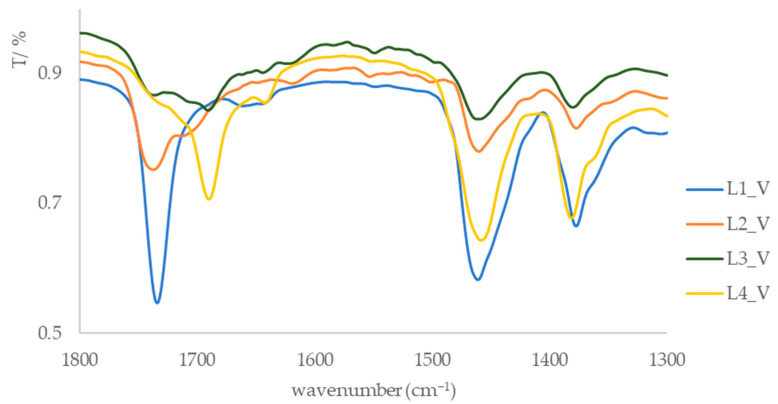
IR spectra of leaf fractions from the island of Vis in the range of 1800–1300 cm^−1^.

Absorption bands between 1721 and 1735 cm^−1^, attributed to the carbonyl groups of carboxylic acids and esters, are also observed in the spectra of the extracts (Figure 4 and Figure 5). In the spectrum of fraction L1, no hydroxyl group band was detected; however, the presence of an absorption band at 1733 cm^−1^ suggests the existence of an ester bond in the isolated compounds. Additionally, absorption bands in the 1680–1700 cm^−1^ range are consistent with conjugated carbonyl functionalities commonly found in flavonoid-related compound classes, although definitive structural assignment cannot be made based solely on IR spectra.

In the IR spectra of all fractions, a specific absorption band at approximately 1460 cm^−1^ with medium intensity is observed, corresponding to the bending vibration of the C–H bond in methylene groups (–CH_2_–). Additionally, an absorption band around 1170 cm^−1^ is attributed to the stretching frequency of the C–O bond in ethers, likely associated with methoxy groups. These observations indicate the presence of phenolic compounds across all fractions, as well as differences in the chemical composition of the compounds in individual fractions. Strong absorption bands in the C–H bond region suggest the presence of both saturated and unsaturated compounds, while bands in the carbonyl group region point to esters or acids. Based on the presence of hydroxyl and carbonyl-related absorption bands in fractions L2 to L4, these fractions can be associated with phenolic-rich compound classes, including flavonoid- and phenolic acid-related structures previously reported in *A. unedo* leaves [29]. However, FTIR spectroscopy provides qualitative information only, and compound-level identification cannot be confirmed without complementary chromatographic or spectrometric techniques.

### 3.2. Effects of Geographical Location, Extraction Technique, and Solvent Type on Polyphenol Recovery and Antioxidant Capacity in A. unedo L. Leaves

The results of the descriptive statistical analysis provide valuable insights into the variability and central tendencies of the bioactive and antioxidant parameters measured across the 36 samples (Table 5). Here, CV% summarizes the overall dispersion of values across all extraction conditions (global variability), while within-extract analytical variability was estimated from triplicate measurements and is not what Table 5 reports. The Total Phenolic Content (TPC) exhibited the highest mean value (55.94 mg GAE g^−1^) among all parameters, accompanied by a notably high standard deviation and coefficient of variation (CV% = 97.09), indicating substantial heterogeneity within the dataset. The wide confidence interval (CI: 37.56–74.32 mg g^−1^) further supports the diversity in phenolic content, likely due to environmental or genetic factors influencing secondary metabolite production [30]. For instance, studies have reported TPC values ranging from 94.51 ± 0.08 to 141.72 ± 0.56 mg GAE g^−1^ extract, depending on the solvent used [31]. Another study reported an average TPC of 61.95 mg GAE g^−1^ dry weight in *A. unedo* leaf extracts [32]. The variability in TPC underscores the importance of optimizing all variable parameters to maximize the yield of bioactive compounds from *A. unedo* leaves. Factors such as geographical location, extraction technique and extraction solvent type should be carefully considered to enhance the efficiency of phenolic extraction. Understanding these variables is essential for the effective utilization of *A. unedo* leaf extracts in industry applications, where high phenolic content is often associated with potent antioxidant properties.

Hydroxycinnamic acids (HCA) and flavonols (FL) followed similar trends with mean values of 15.17 mg g^−1^ and 10.76 mg g^−1^, and CVs of 0.87 and 0.83, respectively. These high CVs suggest considerable diversity in the accumulation of secondary metabolites, potentially driven by differential gene expression and metabolic fluxes under various growing conditions [33]. The mean values fall within the expected ranges reported for diverse cultivars and environmental settings [34]. The content of condensed tannins (CT) was also marked by high variability (mean = 18.38 mg g^−1^; CV = 0.90), supporting the notion that proanthocyanidin biosynthesis is highly responsive to both endogenous and exogenous stimuli [35]. The wide range between the minimum and maximum values for CT (2.19–56.26 mg g^−1^) emphasizes the importance of selection for genotypes with desirable phytochemical profiles. For instance, a study reported average contents of 24.81 ± 0.24 mg 100 g^−1^ for hydroxycinnamic acids, 44.23 ± 0.40 mg 100 g^−1^ for condensed tannins, and 12.06 ± 0.18 mg 100 g^−1^ for flavonols in *A. unedo* fruit extracts [36]. These findings suggest that hydroxycinnamic acids and condensed tannins are more abundant in *A. unedo* leaf and fruit extracts compared to flavonols, which are present in smaller quantities.

In terms of antioxidant activity, ABTS radical scavenging activity had the highest mean (115.71 mmol TE 100 g^−1^), with a wide range (4.90–310.25 mmol TE 100 g^−1^) and substantial variation (CV = 0.81). The FRAP assay also indicated notable antioxidant potential (mean = 72.07 mmol TE 100 g^−1^), though with slightly lower variability (CV = 0.74) compared to ABTS. These results suggest that *A. unedo* leaf extracts possess strong and variable antioxidant capacity, potentially linked to their phenolic composition. The high variability among samples underlines the importance of selecting appropriate harvest conditions and genetic material for functional applications. In Table 5, SD and CV describe the variability among samples/conditions, while the 95% CI indicates the precision (confidence) of the mean estimate. CV% in Table 5 represents the global coefficient of variation computed across all independent extracts/conditions (n = 36), i.e., overall variability across the full dataset rather than within-condition analytical variability.

Table 5 was rebuilt from raw outputs with consistent alignment and complete fields. Descriptive statistics are reported across n = 36 independent extracts (18 conditions × 2 extraction replicates). In order to clearly evaluate the effects of the sources of variation (growing location, extraction technique and solvent type) on the content of bioactive compounds and antioxidant capacity in the extracts studied, a statistical analysis was also performed (Table 6 and Table 7). For clarity, ANOVA results are reported as main effects (LC, TE, ES); potential interaction effects were not included in the primary ANOVA tables and should be explored in future work.

High global CV (Table 5) can coexist with relatively narrow CIs of group means (Table 6) because CV reflects pooled dispersion across all conditions, whereas CI reflects mean precision within groups. The results show that the leaves from Vis had significantly higher values of TPC (62.00 mg GAE g^−1^), CT (21.80 mg CE g^−1^) and slightly higher HCA and FL values compared to Mali Lošinj. This suggests that the environmental or microclimatic conditions on Vis may promote the biosynthesis or accumulation of certain phenolic compounds. The differences in CT and TPC between Mali Lošinj and Vis indicate that geographical origin may be an important determinant of the phytochemical profile of *A. unedo*. The higher content of bioactive compounds in plants from southern geographical locations (e.g., Vis) compared to plants from northern locations (e.g., Mali Lošinj), can generally be attributed to several important environmental and ecological factors [37,38,39]. In summary, the combination of higher solar radiation, longer growing seasons, warmer temperatures, and greater environmental stress in southern geographical locations typically promotes the production of bioactive compounds in plants. These bioactive compounds are often produced as part of plant’ defense mechanisms against environmental stressors, resulting in higher concentrations of bioactive compounds in plants from southern regions compared to those from northern regions [40,41].

No statistically significant differences were found between the three extraction techniques—conventional, Soxhlet, and ultrasound-assisted extraction for any polyphenolic compounds measured. However, ultrasound-assisted extraction resulted in the highest TPC (61.25 mg GAE g^−1^) followed by conventional and Soxhlet extraction. Although not statistically different, these trends suggest that ultrasound slightly improves the extraction of polyphenols due to better disruption of cell walls and shorter extraction times [42]. In Table 6, SD/CV represent data spread across groups, whereas the 95% CI reflects confidence in the group mean (mean precision) and should not be interpreted as sample-to-sample variability.

The choice of solvent had the greatest influence on the recovery of the compounds. Extraction with 70% ethanol resulted in significantly higher concentrations of all bioactive compounds compared to distilled water and ethyl acetate (*p* ≤ 0.05). For example, TPC reached 125.27 mg GAE g^−1^ with 70% ethanol, compared to only 37.46 mg GAE g^−1^ with water and negligible 5.09 mg GAE g^−1^ with ethyl acetate. A similar trend was observed for HCA, FL and CT. These results are consistent with the polarity of the solvents used: ethanol–water mixtures are known to be particularly efficient in extracting a wide range of polyphenols due to their medium polarity and good tissue penetration. In addition, distilled water proved to be a more effective extraction solvent than ethyl acetate, yielding on average 4.3 times more. Distilled water is more polar than 70% ethanol as water is a highly polar molecule due to its hydrogen bonding and dipolar nature. In contrast, 70% ethanol is a mixture where the presence of ethanol (which has both polar and nonpolar moieties) reduces its overall polarity compared to pure water [43]. Ethyl acetate is considered an environmentally friendly solvent due to its low toxicity, biodegradability, and environmental safety, as well as its efficiency in extraction processes and the possibility of using renewable resources for its production. These factors contribute to its reputation as a more sustainable alternative to other solvents [44]. However, due to its moderate polarity, it is well suited for the extraction of moderately non-polar compounds. The results obtained therefore confirm that the extracts from the leaves of *A. unedo* contain bioactive phytochemicals of different polarity, with polar compounds typically found in the aqueous fraction, while less polar or hydrophobic compounds are found in the organic solvent fractions. As a result, 70% ethanol was identified as the best solvent.

The results presented in Table 7 demonstrate that antioxidant capacity of *A. unedo* L. leaf extracts, as measured by FRAP and ABTS assays, was significantly influenced by geographical location, extraction technique, and solvent type (*p* ≤ 0.05). Notably, samples from Vis exhibited higher ABTS activity compared to those from Mali Lošinj, while FRAP values did not differ significantly between the two locations, suggesting potential variation in extractable antioxidant compounds affected more by ABTS-sensitive radical scavenging mechanisms. Among extraction techniques, conventional extraction yielded the highest antioxidant values in both assays, particularly in ABTS, indicating its efficiency in preserving or releasing bioactive compounds compared to Soxhlet and ultrasound-assisted methods. The lower performance of Soxhlet and ultrasound techniques may be attributed to thermal degradation or insufficient disruption of plant matrices under the tested conditions [45]. Solvent type was the most influential factor, with 70% ethanol significantly outperforming both distilled water and ethyl acetate in both assays. Aqueous ethanol solutions, such as 70% ethanol, combine water’s polarity with ethanol’s ability to solubilize both polar and non-polar compounds, making them particularly effective for extracting a wide range of bioactive compounds [9]. Conversely, ethyl acetate extracts displayed minimal antioxidant activity, suggesting that this solvent is ineffective for extracting the major antioxidant constituents from *A. unedo* leaves. As a medium-polarity solvent, ethyl acetate is especially effective for extracting less polar compounds. Its selectivity is advantageous for isolating specific bioactive components like carotenoids and flavonoids from non-polar matrices [46]. Overall, the findings highlight that comparative evaluation of extraction parameters, particularly solvent choice, is critical for maximizing the antioxidant potential of *A. unedo* leaf extracts.

#### 3.2.1. Correlation Analysis of Bioactive Compounds and Antioxidant Activities

**Figure 6:**
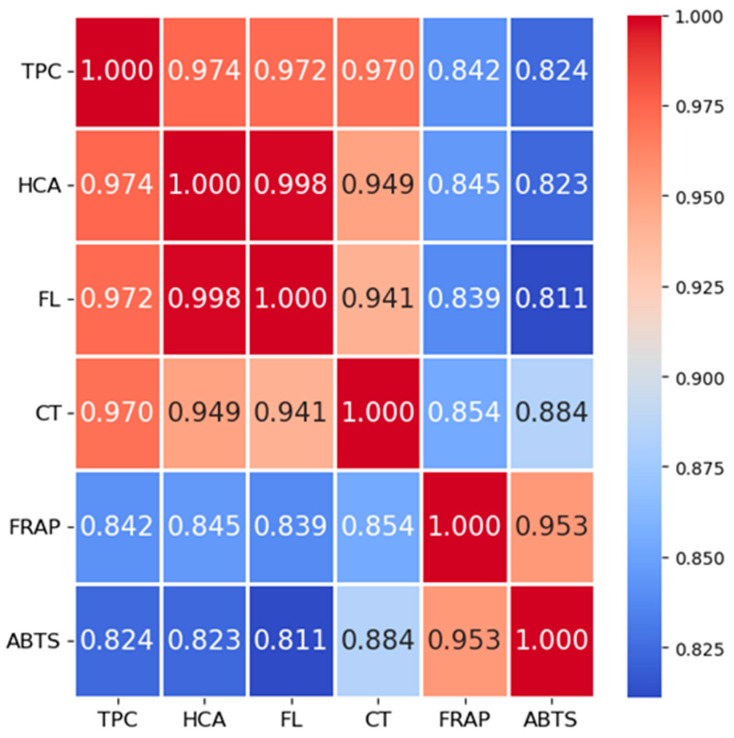
Pearson correlation matrix among total phenolic content (TPC), hydroxycinnamic acids (HCA), flavonols (FL), condensed tannins (CT), and antioxidant activity assays (FRAP and ABTS) (Values range from −1 (perfect negative correlation) to +1 (perfect positive correlation)).

The correlation matrix presented in Figure 6 illustrates the relationships among the dependent variables, including total phenolic content (TPC), hydroxycinnamic acids (HCA), flavonols (FL), condensed tannins (CT), and antioxidant activity measured by FRAP and ABTS assays. Overall, a strong positive correlation was observed among most variables, suggesting that phenolic composition significantly contributes to antioxidant potential. TPC exhibits a very high correlation with HCA (r = 0.97), FL (r = 0.97), and CT (r = 0.97), which is expected given that these compounds constitute major subclasses of phenolic compounds. This further indicates that increases in TPC are generally reflective of increases in specific phenolic subgroups. Similarly, FL shows strong correlations with both HCA (r = 0.98) and CT (r = 0.94), underscoring the biochemical interrelationships and possible co-extraction or co-localization of these compounds in the samples studied. HCA and CT are also positively correlated (r = 0.95), reinforcing this observation.

Antioxidant assays (FRAP and ABTS) demonstrate moderately strong correlations with phenolic components. FRAP shows particularly strong correlation with TPC (r = 0.84), CT (r = 0.85), and ABTS (r = 0.95), indicating that reducing capacity is closely associated with total phenolic content and condensed tannin levels. ABTS shows its highest correlation with FRAP (r = 0.95), followed by CT (r = 0.88), suggesting that both assays capture overlapping but slightly distinct aspects of antioxidant activity. Notably, ABTS correlates slightly less with HCA and FL (r = 0.82 and r = 0.81, respectively), which may reflect differences in the radical scavenging mechanisms detected by the ABTS assay compared to the phenolic content contributions of these specific compounds.

In summary, the matrix underscores the integral role of phenolic compounds—particularly TPC, CT, and FL, in contributing to antioxidant capacity. These strong inter-correlations support the use of phenolic content as a proxy indicator for antioxidant potential in future studies. However, the strong correlations observed among TPC, HCA, FL, and CT partly reflect the conceptual and methodological overlap of these spectrophotometric assays, as they represent interrelated phenolic subclasses rather than fully independent chemical measures. This correlation structure also implies that simple linear baselines may already capture a substantial fraction of the variance for several targets when using TPC as the primary numerical predictor. Therefore, the added value of more flexible ML models should be interpreted in relation to these baselines.

#### 3.2.2. Results: Regression Model Performance

The evaluation includes an analysis of various regression models used to predict HCA, FL, CT, ABTS, and FRAP. The outcomes of the highest-ranked feature-based prediction models on the test set are summarized in Table 8. Target variables were not normalized/scaled. RMSE and MAE in Table 8 are reported in the original measurement units of each response variable, while R^2^ is unitless. Given the strong correlations between TPC and several targets (Figure 6), we additionally evaluated simple baselines (Linear Regression) and regularized linear models (Ridge, Lasso, ElasticNet) using the same predictors (TPC + LC/TE/ES). In this dataset, linear/regularized baselines achieved high predictive performance for CT (Test R^2^ = 0.94) and moderate to high performance for ABTS (Test R^2^ = 0.79), indicating that a large portion of the signal can be captured by simple relationships. For HCA and FL, regularization improved generalization compared to ordinary least squares (Test R^2^ = 0.82 for HCA and =0.74 for FL), while FRAP achieved Test R^2^ = 0.85. These results suggest that more complex models may provide incremental gains primarily by capturing mild non-linearities and interactions beyond the dominant TPC-driven linear trend (Baseline results are provided in Table S1). Given the very strong correlations between TPC and several targets, we compared complex ML models against simple linear baselines (Table S1). The baseline results show that a substantial portion of the predictive signal is captured by linear relationships using TPC and categorical descriptors, while more complex models provide at most incremental gains within this dataset.

The complex models in Table 8 should be interpreted as exploratory fits, and their apparent improvements over linear baselines should be validated on independent datasets before any broader generalization. If accurate and robust models can be constructed using TPC and basic categorical descriptors, they could help estimate HCA, FL, CT, ABTS, and FRAP within this experimental setting, thereby reducing the number of time-consuming and costly chemical assays needed during early-stage screening. This approach is intended as a preliminary screening tool to support rapid comparative assessment of extraction conditions, rather than as a substitute for detailed chemical analyses.

Although high coefficients of determination were obtained for several target variables, these results reflect model performance within the investigated experimental space.

The results show that tree-based models, especially the Decision Tree Regressor (DTR) and the Gradient Boosting Regressor (GBR), achieved high prediction accuracy for most of the target variables, while the AdaBoost Regressor (ABR) showed relatively lower performance for ABTS prediction which may reflect that ABTS is influenced by additional chemical descriptors beyond TPC and the categorical extraction factors, rather than being fully captured by increasing model complexity alone.

The Decision Tree Regressor demonstrated superior performance in predicting HCA and FL, achieving R^2^ values of 0.9744 and 0.9472, respectively. These results indicate a strong linear or nonlinear relationship between TPC and these target compounds, which the Decision Tree model effectively captured. The low Mean Absolute Error (MAE) values for both predictions (0.0277 for HCA and 0.0363 for FL) further confirm the accuracy of the model. For CT and FRAP, the Gradient Boosting Regressor performed best, with R^2^ values of 0.9470 and 0.9147, respectively. While slightly lower than the Decision Tree Regressor in HCA and FL prediction, the Gradient Boosting model provided stable and reliable predictions. The slightly increased Root Mean Squared Error (RMSE) values (0.0487 for CT and 0.0660 for FRAP) indicate a moderate level of residual variability, which could be due to the complexity of these chemical interactions. In contrast, the AdaBoost Regressor performed relatively poorly in predicting ABTS, with an R^2^ of only 0.6768. The lower accuracy of AdaBoost in this case could be due to the nature of the dataset, where the relationship between TPC and ABTS is more complex and potentially nonlinear, suggesting that additional descriptors beyond TPC and the categorical factors may be needed for more robust ABTS estimation. The training times (TT) for the different models indicate that the Decision Tree Regressor was the fastest (0.0262–0.0288 sec), making it an efficient choice for fast predictions. Gradient Boosting Regressor required slightly more computational time (0.0712 sec), while AdaBoost had the longest processing time (0.1500 sec). Given its lower predictive power, the use of AdaBoost for ABTS modeling may not be justified in practical applications.

The high predictive accuracy of the Decision Tree and Gradient Boosting models for HCA, FL, CT, and FRAP suggests that these antioxidant properties can be estimated within this dataset, indicating that the relationships are strong in this experimental setting. This finding supports the possibility of reducing the need for direct analytical measurement of these parameters, as their values can be derived with high confidence from TPC. In contrast to other target variables, ABTS prediction exhibited notably lower model performance, highlighting limitations of using TPC as a universal proxy for antioxidant activity. Unlike FRAP, which is closely associated with reducing power and total phenolic content, the ABTS assay captures multiple radical-scavenging mechanisms influenced by compound structure, molecular weight, and synergistic interactions among antioxidants. These factors are not fully represented by TPC alone, which likely contributed to reduced predictive accuracy. This finding underscores the need for incorporating additional chemical descriptors or complementary analytical parameters when modeling complex antioxidant responses. Overall, the tree-based models showed strong predictive abilities for most antioxidant-acting compounds, with the Decision Tree Regressor standing out for HCA and FL prediction, and the Gradient Boosting Regressor providing stable performance for CT and FRAP.

It should be noted that spectrophotometric assays, such as the Folin–Ciocalteu method for TPC determination, have inherent limitations. The Folin–Ciocalteu reagent is not fully specific to phenolics and can react with other reducing compounds, while overlaps between different phenolic subclasses can reduce analytical selectivity. These factors should be considered when interpreting the results, particularly because TPC and related spectrophotometric parameters were used as targets in the machine learning analysis. Despite these limitations, spectrophotometric assays provide a rapid and broadly accessible measure of overall phenolic content, which is suitable for initial exploratory modeling and comparative evaluations.

The results suggest that machine learning could help prioritize conditions for further testing by reducing the number of chemical analyses required, allowing potential applications in food science and natural product research.

From a practical perspective, the proposed models may serve as preliminary screening tools that support rapid comparative assessment of extraction conditions, rather than as substitutes for detailed chemical analysis. In this context, machine learning contributes primarily to guiding decision-making and narrowing experimental space, rather than defining optimal conditions in a strict comparative evaluation framework. While the results suggest potential relevance for industrial valorization of *A. unedo* leaves, additional studies addressing scale-up, process robustness, and economic feasibility are required before industrial implementation can be considered.

Limitations and scope. Given the small dataset (n = 36) and the absence of external or structured cross-location validation (e.g., leave-one-location-out), the ML results should be interpreted as exploratory and specific to the experimental space investigated. Accordingly, the proposed models are intended to support early-stage, low-cost screening based on TPC and categorical extraction descriptors rather than to claim broad predictive generalization.

## 4. Conclusions

This study demonstrates that the bioactive profile and antioxidant potential of *Arbutus unedo* leaf extracts are significantly affected by extraction parameters, particularly solvent type and geographical origin. Among the methods tested, 70% ethanol proved to be the most effective solvent, extracting the highest levels of phenolic compounds and antioxidant activity. Leaves from the island of Vis exhibited superior phenolic content and antioxidant activity compared to those from Mali Lošinj, emphasizing the role of environmental factors. The IR spectra of the purified fractions obtained by column chromatography show the chemical diversity of the isolated components.

The ML analysis supports the use of TPC and basic extraction descriptors as a context-specific, low-cost screening aid within the investigated experimental space. Broader generalization requires validation on independent datasets and structured cross-location testing.

Overall, the integration of green extraction strategies with data-driven analytical support offers a promising approach for the comparative evaluation and valorization of underutilized plant resources. Future research should focus on expanding the experimental design and validating the proposed models to enable broader applicability and informed process screening. In line with this, we highlight the need to incorporate additional and more chemically selective descriptors to improve model robustness and interpretability.

## Figures and Tables

**Table 1:** Mean extract mass and extraction yield of leaf extracts from plants collected on the islands of Vis and Mali Lošinj following conventional extraction (CE).

Solvent	Mean Mass ± SD (mg)	Yield ± SD (%*w*/*w*)	Mean Mass ± SD (mg)	Yield ± SD (%*w*/*w*)
	Vis		Mali Lošinj	
Water	520.00 ± 5.00	10.40 ± 0.01	511.33 ± 3.51	10.23 ± 0.07
Ethanol 70%	2663.00 ± 2.00	53.26 ± 0.04	2067.70 ± 2.50	41.35 ± 0.05
Ethyl acetate	87.00 ± 3.00	1.74 ± 0.06	65.00 ± 2.00	1.30 ± 0.40

Yield values are reported as mean ± SD of three repeated mass measurements used to compute yield for each extract (n = 3); these are instrumental repeats and were not treated as independent extraction replicates.

**Table 2:** Mean extract mass and extraction yield of leaf extracts from plants collected on the islands of Vis and Mali Lošinj following Soxhlet extraction (SE).

Solvent	Mean Mass ± SD (mg)	Yield ± SD (%*w*/*w*)	Mean Mass ± SD (mg)	Yield ± SD (%*w*/*w*)
	Vis		Mali Lošinj	
Water	212.83 ± 2.75	4.26 ± 0.06	207.47 ± 0.85	4.15 ± 0.02
Ethanol 70%	1345.7 ± 6.03	26.91 ± 0.12	1512.70 ± 5.49	30.25 ± 0.11
Ethyl acetate	46.33 ± 2.52	0.93 ± 0.05	75.07 ± 1.13	1.50 ± 0.02

Yield values are reported as mean ± SD of three repeated mass measurements used to compute yield for each extract (n = 3); these are instrumental repeats and were not treated as independent extraction replicates.

**Table 3:** Experimental plan for the extraction of BACs from *A. unedo* leaves.

Sample ID	Geographical Location	Extraction Type	Solvent
1	Mali Lošinj	CE	Water
2			Ethanol 70%
3			Ethyl acetate
4		SE	Water
5			Ethanol 70%
6			Ethyl acetate
7		UAE	Water
8			Ethanol 70%
9			Ethyl acetate
10	Vis	CE	Water
11			Ethanol 70%
12			Ethyl acetate
13		SE	Water
14			Ethanol 70%
15			Ethyl acetate
16		UAE	Water
17			Ethanol 70%
18			Ethyl acetate

**Table 4:** Relative mass share (%) of fractions obtained after column chromatography of leaf extracts from the islands of Vis and Mali Lošinj.

Eluens	Leaves Share (%)	
	Vis	Mali Lošinj
Dichloromethane	19.7	9.6
Dichloromethane:Ethanol 10:0.5	7.5	9.8
Dichloromethane:Ethanol 10:1	27.6	66.2
Ethanol	45.2	14.4

Note: Share (%) represents the mass percentage of each fraction relative to the total mass of isolated dominant components (∑ fractions = 100%).

**Table 5:** Descriptive statistics for bioactive compounds and antioxidant capacity in *A. unedo* leaf extracts V = Variable; TPC—Total phenolic content (mg GAE g^−1^); HCA—Hydroxycinnamic acids (mg CAE g^−1^); FL—Flavonols (mg QE g^−1^); CT—Condensed tannins (mg CE g^−1^); FRAP—Results from FRAP assay (mmol TE 100 g^−1^); ABTS—Results from ABTS assay (mmol TE 100 g^−1^); Mean—Average value; SD—Standard deviation; Min/Max = minimum/maximum; CV%—Coefficient of variation; CI_lower/CI_upper = lower/upper bounds of the 95% confidence interval for the mean.

V	N	Mean	SD	CV%	Min	Max	Median	Q1	Q3	95% CI_Low	95% CI_High
TPC	36	55.95	54.32	97.09	2.82	156.28	30.28	5.88	116.79	37.57	74.32
HCA	36	15.17	13.39	88.25	1.36	39.41	11.67	3.18	28.06	10.64	19.7
FL	36	10.76	9.05	84.12	0.91	26.52	8.1	2.86	18.53	7.7	13.82
CT	36	18.39	16.84	91.59	2.19	56.26	13.35	3.29	31.12	12.69	24.08
FRAP	36	72.06	54.12	75.11	2.2	150	88.9	7.32	121	53.75	90.37
ABTS	36	115.72	94.87	81.98	4.9	310.3	126	13.48	191.12	83.62	147.82

CV% represents the global coefficient of variation calculated across all independent extracts/conditions (n = 36), i.e., overall variability across the full dataset.

**Table 6:** Effect of geographical location, extraction technique, and solvent type on the recovery of polyphenolic compounds from *A. unedo* leaf extracts.

V	Level	TPC			HCA			FL			CT		
		Mean ± CI	SD	Min–Max	Mean ± CI	SD	Min–Max	Mean ± CI	SD	Min–Max	Mean ± CI	SD	Min–Max
GL	ML	49.88 ± 0.44 ^a^	51.05	2.82–130.03	14.32 ± 0.16 ^a^	14.46	1.35–39.41	10.29 ± 0.12 ^a^	9.86	0.91–26.52	14.96 ± 0.19 ^a^	14.18	2.19–40.70
	V	62.00 ± 0.44 ^b^	58.23	2.91–156.28	16.01 ± 0.16 ^a^	12.59	2.32–36.47	11.22 ± 0.12 ^a^	8.42	2.09–25.19	21.80 ± 0.19 ^b^	18.92	2.62–56.26
TE	CE	58.05 ± 0.54 ^a^	63.53	4.57–156.28	15.65 ± 0.20 ^a^	15.91	2.33–39.41	10.97 ± 0.14 ^a^	10.64	2.09–26.41	20.28 ± 0.23 ^a^	21.11	2.19–56.26
	SE	48.52 ± 0.54 ^a^	54.39	5.84–125.80	14.72 ± 0.20 ^a^	14.85	1.35–35.93	10.57 ± 0.14 ^a^	10.31	0.91–26.52	16.46 ± 0.23 ^a^	15.51	2.86–40.60
	UAE	61.25 ± 0.54 ^a^	47.92	2.82–123.78	15.12 ± 0.20 ^a^	9.8	3.15–28.48	10.72 ± 0.14 ^a^	6.39	2.72–19.34	18.40 ± 0.23 ^a^	14.43	3.38–43.67
S	Water	37.46 ± 0.54 ^b^	25.56	5.89–83.32	10.04 ± 0.21 ^b^	5.62	1.35–16.18	7.18 ± 0.14 ^b^	4.08	0.91–11.91	12.17 ± 0.23 ^b^	5.56	2.86–18.67
	Ethanol 70%	125.27 ± 0.54 ^c^	17.05	97.27–156.28	32.40 ± 0.20 ^c^	5.04	23.86–39.41	22.34 ± 0.14 ^c^	3.63	16.80–26.52	39.78 ± 0.23 ^c^	8.82	29.28–56.26
	Ethyl acetate	5.09 ± 0.54 ^a^	2.18	2.82–9.35	3.05 ± 0.20 ^a^	0.69	2.32–4.00	2.74 ± 0.14 ^a^	0.57	2.09–3.69	3.19 ± 0.23 ^a^	0.68	2.19–4.38

Different superscript letters within a factor indicate significant differences (Tukey’s HSD, *p* < 0.05). SD and Min–Max describe within-group dispersion among independent extracts, whereas the 95% CI reflects the precision of the group mean. V= Variable; GL = Geographical location; TE = Extraction technique; S = Solvent; ML = Mali Lošinj; V = Vis; Results are expressed as mean ± 95% CI. Values represented with different letters are statistically different at *p* ≤ 0.05. TPC—Total phenolic content (mg GAE g^−1^); HCA—Hydroxycinnamic acids (mg CAE g^−1^); FL—Flavonols (mg QE g^−1^); CT—Condensed tannins (mg CE g^−1^). CI values are computed from independent extracts (not analytical triplicates).

**Table 7:** The impact of geographical location, extraction technique, and solvent type on the antioxidant capacity from *A. unedo* leaf extracts.

V		FRAP	ABTS
GL	ML	64.72 ± 0.52 ^a^	92.42 ± 2.57 ^a^
	V	79.41 ± 0.52 ^a^	138.99 ± 2.57 ^b^
ET	CE	80.26 ± 0.64 ^a^	142.37 ± 3.14 ^a^
	SE	68.92 ± 0.64 ^a^	112.72 ± 3.14 ^a^
	UAE	67.02 ± 0.64 ^a^	92.04 ± 3.14 ^a^
S	Water	85.04 ± 0.64 ^b^	127.89 ± 3.14 ^b^
	Ethanol 70%	125.73 ± 0.64 ^c^	209.83 ± 3.14 ^c^
	Ethyl acetate	5.43 ± 0.64 ^a^	9.41 ± 3.14 ^a^

V = Variable; GL = Geographical location; ET = Extraction technique; S = Solvent; ML = Mali Lošinj; V = Vis; Results are expressed as mean ± 95% CI. Values represented with different letters are statistically different at *p* ≤ 0.05. FRAP—Ferric Reducing Antioxidant Power (mmol 100 g^−1^), ABTS—2,2′-Azino-bis(3-ethylbenzothiazoline-6-sulfonic acid (mmol Trolox equivalents 100 g^−1^).

**Table 8:** Performance metrics of the selected models for predicting HCA, FL, CT, FRAP and ABTS using LC, ES, and TE categories and TPC values.

Predict Variable	Model	MAE	MSE	RMSE	R^2^ (Mean ± SD)	RMSLE	MAPE	TT (sec)
HCA	DTR	0.0277	0.0014	0.0325	0.9744 ± 0.1417	0.0217	0.1599	0.0262
FL	DTR	0.0363	0.0027	0.0424	0.9472 ± 0.2649	0.0270	2.7457	0.0288
CT	GBR	0.0348	0.0047	0.0487	0.9470 ± 0.0233	0.0320	0.3236	0.0712
FRAP	GBR	0.0473	0.0075	0.0660	0.9147 ± 0.0316	0.0414	0.2232	0.0712
ABTS	ABR	0.0819	0.0160	0.1066	0.6768 ± 0.3395	0.0727	4.8114	0.1500

Metrics were computed using k-fold cross-validation with a leakage-free pipeline (pre-processing fit within each training fold only). R^2^ is reported as mean ± SD across folds (unitless). RMSE and MAE are reported in the original units of each response variable.

## Data Availability

The data used to support the findings of this study can be made available by the corresponding authors upon request.

## Supplementary Materials

The following supporting information can be downloaded at: https://www.mdpi.com/article/10.3390/foods15060993/s1, Table S1: Raw extract masses (mg) per replicate for leaf extracts from plants collected on the islands of Vis and Mali Lošinj following conventional extraction (CE); Table S2: Raw extract masses (mg) per replicate for leaf extracts from plants collected on the islands of Vis and Mali Lošinj following Soxhlet extraction (SE); Table S3: *R_f_* values of the four dominant components in leaf extracts obtained by conventional extraction (CE) from Vis and Mali Lošinj using different solvents; Table S4: *R_f_* values of the four dominant components in leaf extracts obtained by Soxhlet extraction (SE) from Vis and Mali Lošinj using different solvents; Figure S1: IR spectra of compounds L1_ML; Figure S2: IR spectra of compounds L2_ML; Figure S3: IR spectra of compounds L3_ML; Figure S4: IR spectra of compounds L4_ML; Figure S5: IR spectra of compounds L1_V; Figure S6: IR spectra of compounds L2_V; Figure S7: IR spectra of compounds L3_V; Figure S8: IR spectra of compounds L4_V.
