# Supplementary material for: Predicting Bioactive Compounds in Arbutus unedo L. Leaves Using Machine Learning: Influence of Extraction Technique, Solvent Type, and Geographical Location
Source: Foods. 2026 Mar 11;15(6):993. doi: 10.3390/foods15060993 (PMC13024744; doi:10.3390/foods15060993)

Table S1. Raw extract masses (mg) per replicate for leaf extracts from plants collected on the islands of Vis and Mali Lošinj following conventional extraction (CE).

Solvent	Replicate	Vis (mg)	Mali Lošinj (mg)
	1	520.00	511.00
Water	2	515.00	508.00
	3	525.00	515.00
	1	2661.00	2065.20
Ethanol 70 %	2	2663.00	2067.70
	3	2665.00	2070.20
	1	90.00	67.00
Ethyl-acetate	2	87.00	63.00
	3	84.00	65.00

Yield calculated using 5000 mg of dry plant material as denominator.

Table S2. Raw extract masses (mg) per replicate for leaf extracts from plants collected on the islands of Vis and Mali Lošinj following Soxhlet extraction (SE).

Solvent	Replicate	Vis (mg)	Mali Lošinj (mg)
	1	210.00	207,50
Water	2	215.50	208,30
	3	213.00	206,60
	1	1340.00	1507.00
Ethanol 70 %	2	1345.00	1512.00
	3	1352.00	1518.00
	1	44.00	74.00
Ethyl-acetate	2	46.00	75.00
	3	49.00	76.20

Yield calculated using 5000 mg of dry plant material as denominator.

Table S3. R_f values of the four dominant components in leaf extracts obtained by conventional extraction (CE) from Vis and Mali Lošinj using different solvents

Island	Solvent	Fraction 1	Fraction 2	Fraction 3	Fraction 4
Vis	Water	0.00	0.461	0.697	0.813
	Ethanol 70 %	0.00	0.475	0.599	0.796
	Ethyl acetate	0.00	0.421	0.578	0.796
Mali Lošinj	Water	0.00	0.435	0.597	0.871
	Ethanol 70 %	0.00	0.407	0.593	0.852
	Ethyl acetate	0.00	0.484	0.656	0.855

Note: In all extracts, four dominant components were consistently observed by TLC. Fractions with identical TLC profiles within the same island were pooled to obtain representative fractions for further analysis.

Table S4. R_f values of the four dominant components in leaf extracts obtained by Soxhlet extraction (SE) from Vis and Mali Lošinj using different solvents

Island	Solvent	Fraction 1	Fraction 2	Fraction 3	Fraction 4
Vis	Water	0.00	0.511	0.651	0.844
	Ethanol 70%	0.00	0.448	0.651	0.854
	Ethyl acetate	0.00	0.343	0.653	0.828
Mali Lošinj	Water	0.00	0.409	0.654	0.739
	Ethanol 70 %	0.00	0.389	0.630	0.796
	Ethyl acetate	0.00	0.371	0.645	0.756

Note: In all extracts, four dominant components were consistently observed by TLC. Fractions with identical TLC profiles within the same island were pooled to obtain representative fractions for further analysis.

Figure S1. IR spectra of compounds L1_ML.

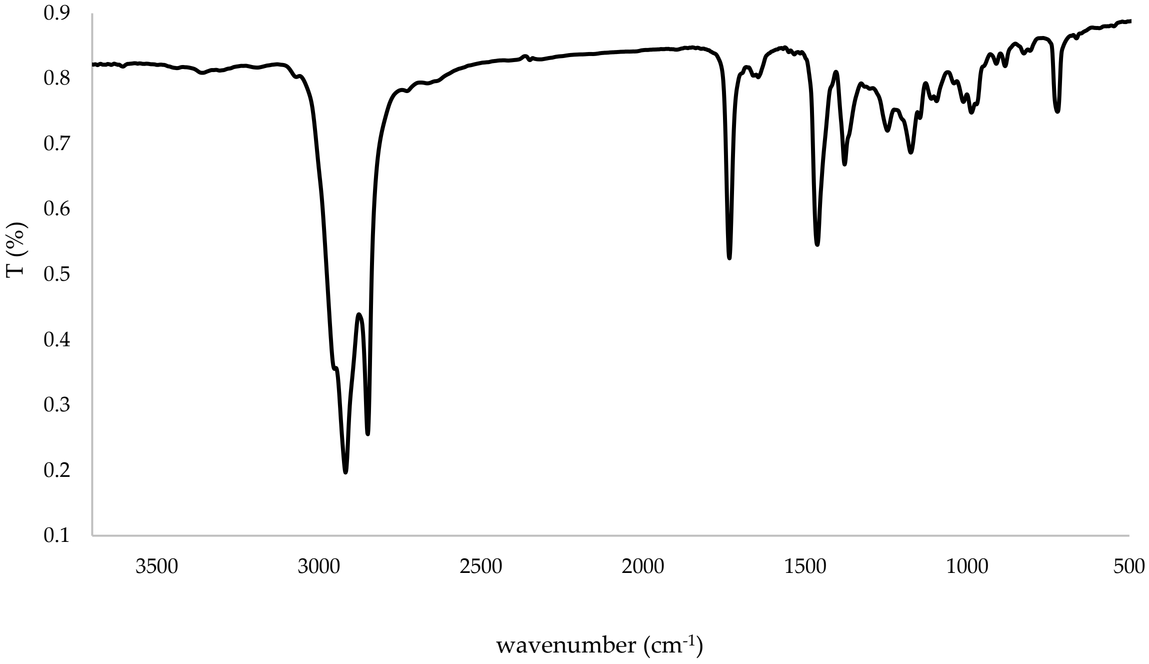

Figure S2. IR spectra of compounds L2_ML.

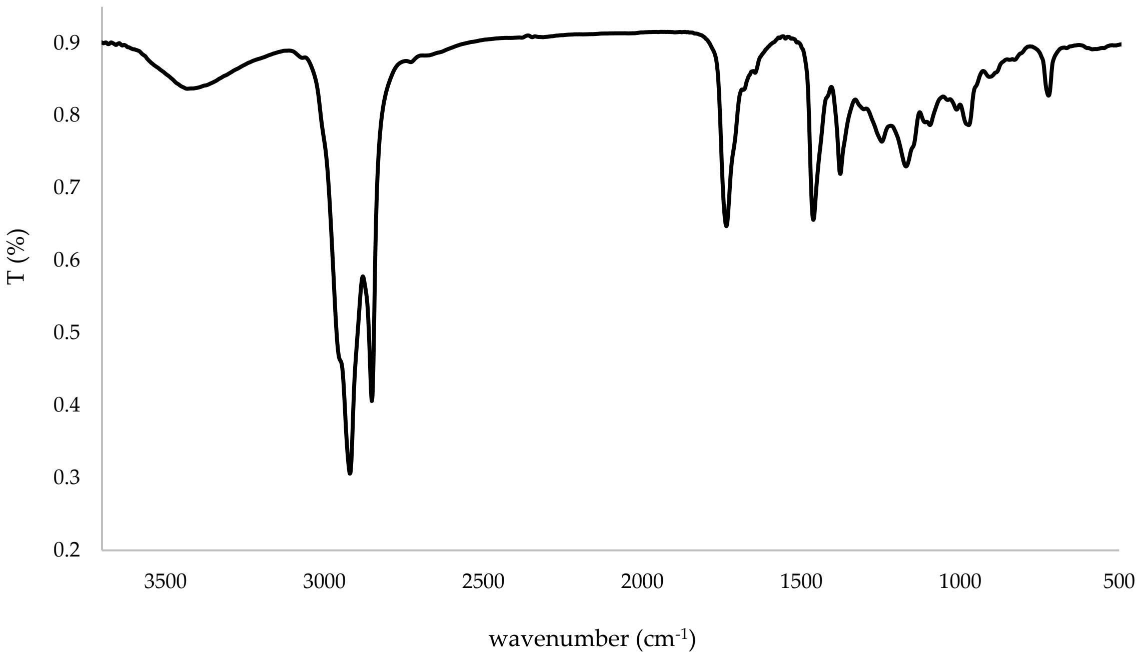

Figure S3. IR spectra of compounds L3_ML.

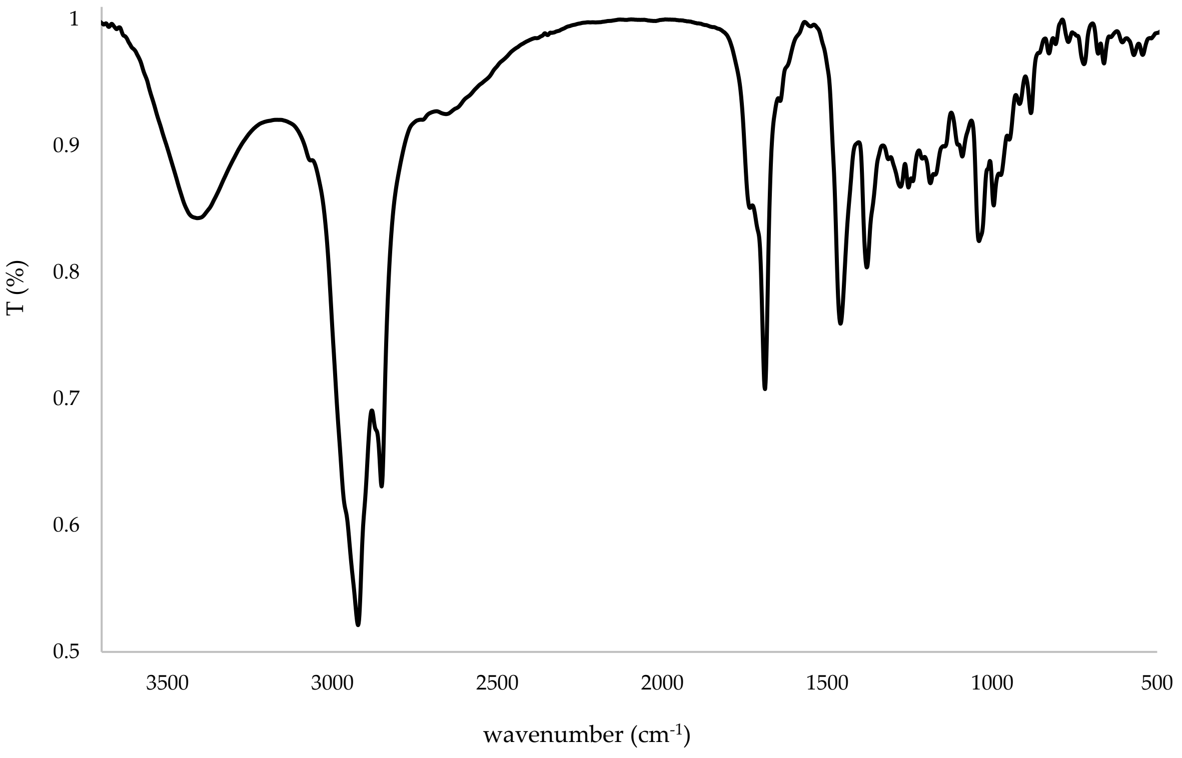

Figure S4. IR spectra of compounds L4_ML.

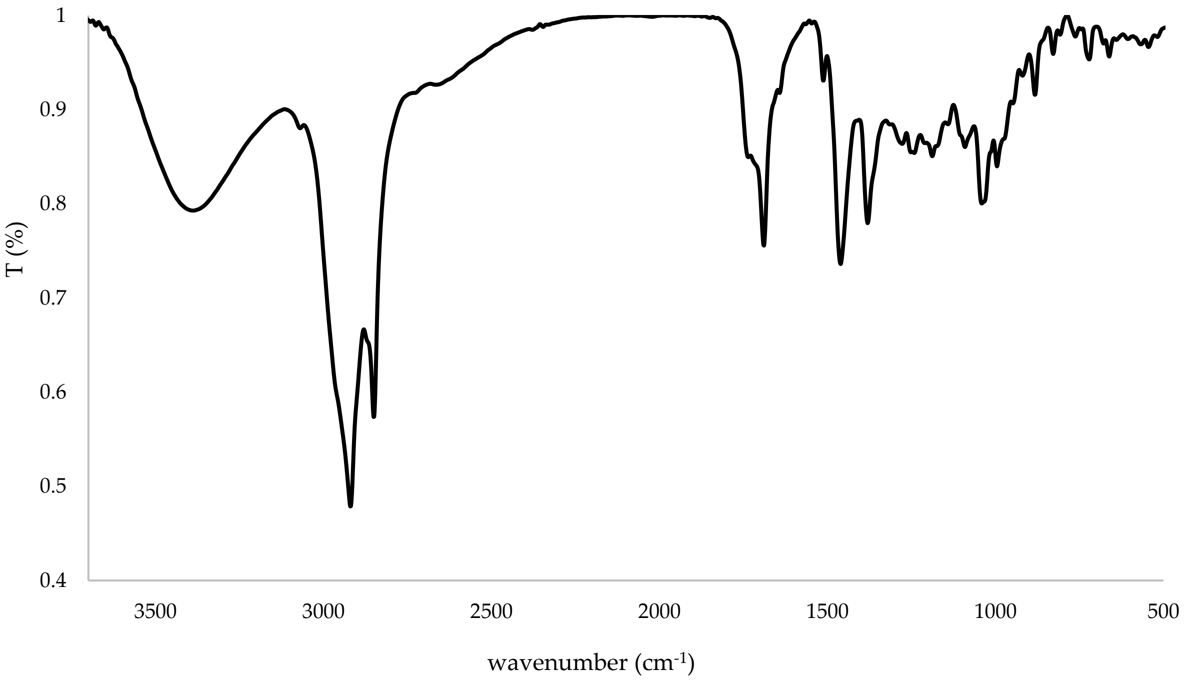

Figure S5. IR spectra of compounds L1_V.

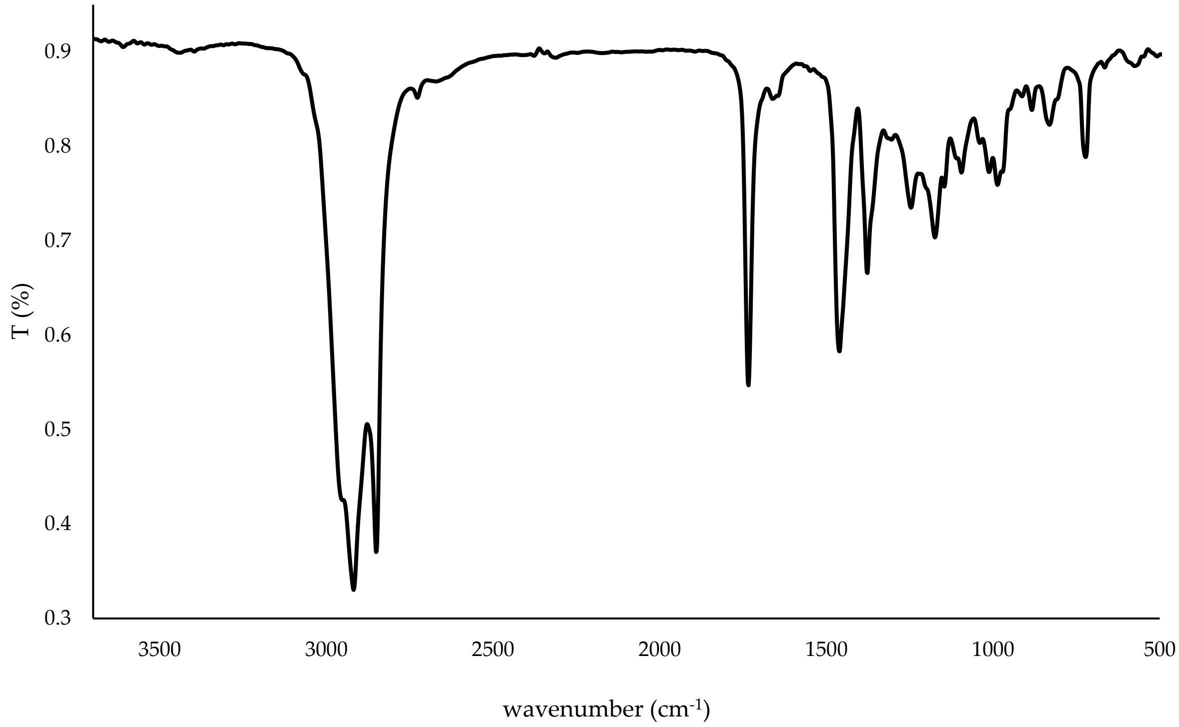

Figure S6. IR spectra of compounds L2_V.

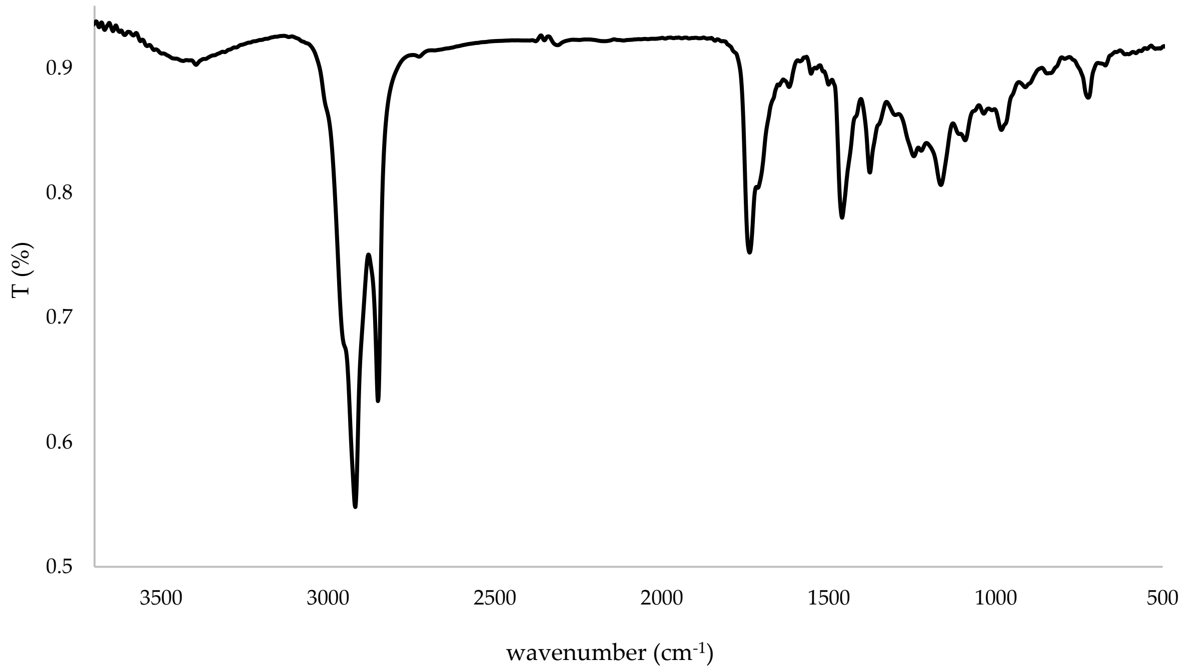

Figure S7. IR spectra of compounds L3_V.

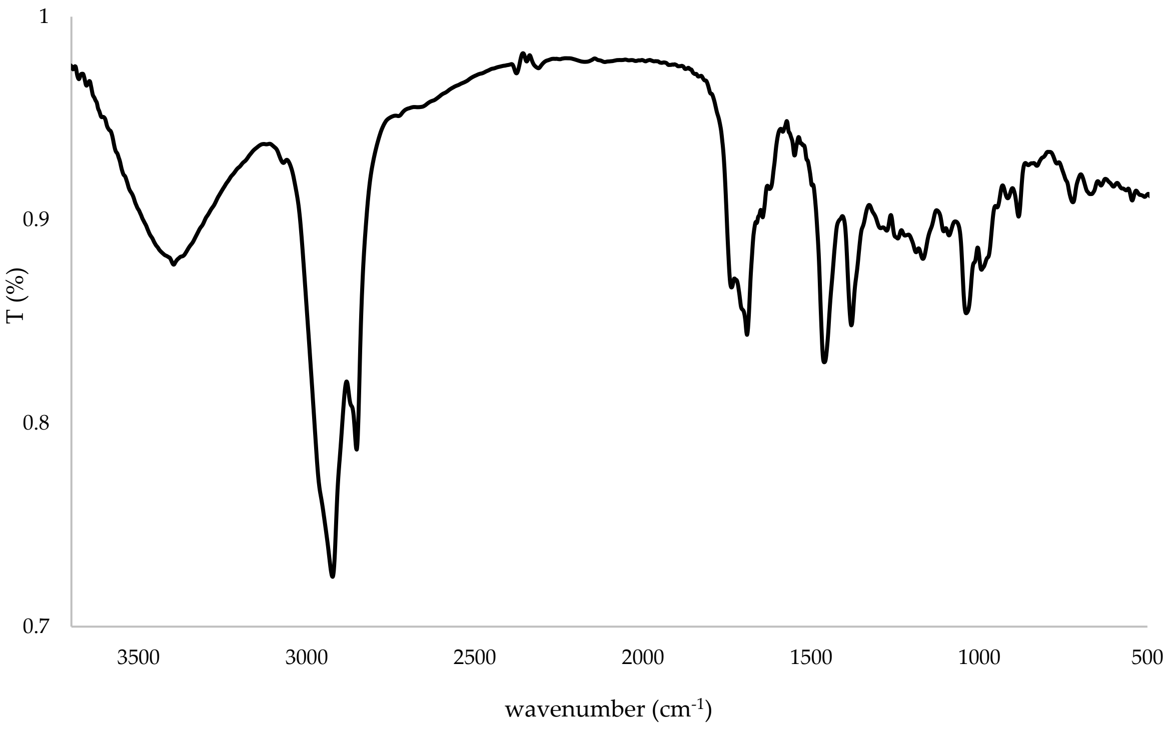

Figure S8. IR spectra of compounds L4_V.

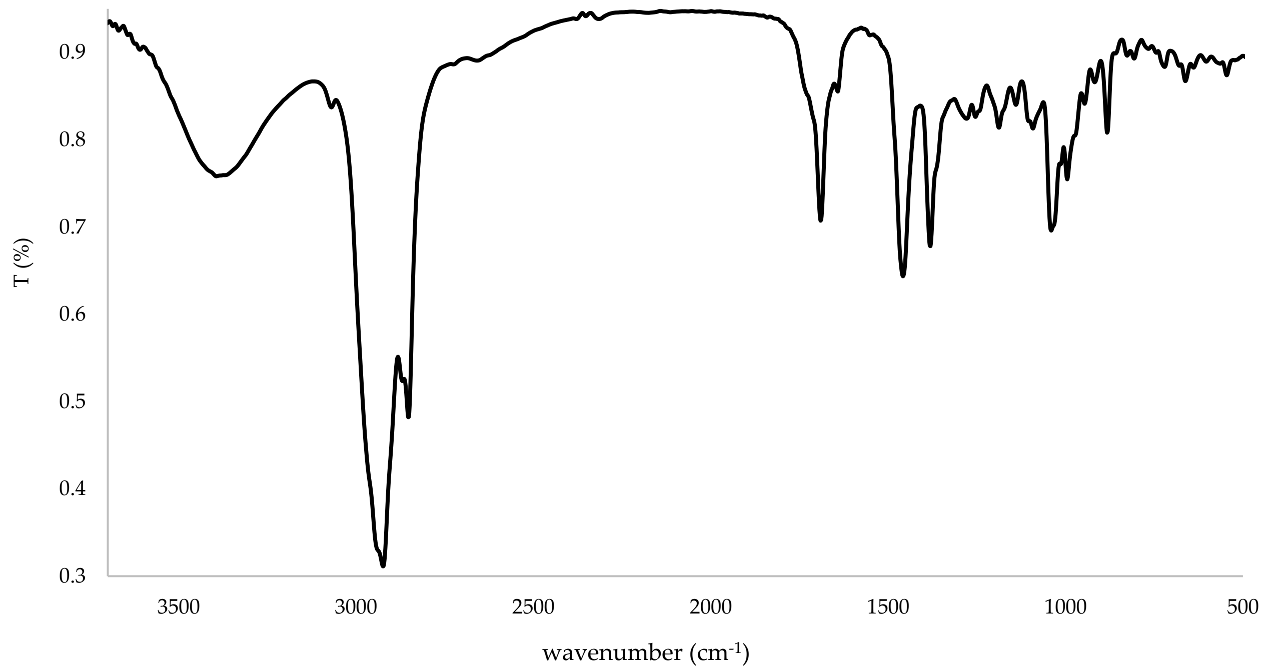

Supplement: Supplementary file 1 [file foods-15-00993-s001.zip › foods-4149142-supplementary.pdf]
